# Infrared Absorption of Laser Patterned Sapphire Al_2_O_3_ for Radiative Cooling

**DOI:** 10.3390/mi16040476

**Published:** 2025-04-16

**Authors:** Nan Zheng, Daniel Smith, Soon Hock Ng, Hsin-Hui Huang, Dominyka Stonytė, Dominique Appadoo, Jitraporn Vongsvivut, Tomas Katkus, Nguyen Hoai An Le, Haoran Mu, Yoshiaki Nishijima, Lina Grineviciute, Saulius Juodkazis

**Affiliations:** 1 Optical Sciences Centre and ARC Training Centre in Surface Engineering for Advanced Materials (SEAM), School of Science, Swinburne University of Technology, Hawthorn, VIC 3122, Australia; danielsmith@swin.edu.au (D.S.); soonhockng@swin.edu.au (S.H.N.); tkatkus@swin.edu.au (T.K.); ale@swin.edu.au (N.H.A.L.); haoranmu@swin.edu.au (H.M.); sjuodkazis@swin.edu.au (S.J.); 2 Laser Research Centre, Physics Faculty, Vilnius University, Saulėtekio Ave. 10, LT-10223 Vilnius, Lithuania; dominyka.stonyte@ff.vu.lt; 3 THz Beamline, ANSTO-Australian Synchrotron, 800 Blackburn Rd, Clayton, VIC 3168, Australia; dominiqa@ansto.gov.au; 4 Infrared Microspectroscopy (IRM) Beamline, ANSTO-Australian Synchrotron, 800 Blackburn Rd, Clayton, VIC 3168, Australia; jitrapov@ansto.gov.au; 5 Department of Electrical and Computer Engineering, Graduate School of Engineering, Yokohama National University, 79-5 Tokiwadai, Hodogaya-ku, Yokohama 240-8501, Kanagawa, Japan; nishijima-yoshiaki-sp@ynu.ac.jp; 6 Institute of Advanced Sciences, Yokohama National University, 79-5 Tokiwadai, Hodogaya-ku, Yokohama 240-8501, Kanagawa, Japan; 7 Institute for Multidisciplinary Sciences, Yokohama National University, 79-5 Tokiwadai, Hodogaya-ku, Yokohama 240-8501, Kanagawa, Japan; 8 PRESTO, Japan Science and Technology Agency (JST), 4-1-8 Honcho, Kawaguchi 332-0012, Saitama, Japan; 9 Center for Physical Sciences and Technology (FTMC), Savanoriu Ave. 231, LT-02300 Vilnius, Lithuania; lina.grineviciute@ftmc.lt; 10 WRH Program International Research Frontiers Initiative (IRFI) Institute of Science Tokyo, Nagatsuta-cho, Midori-ku, Yokohama 226-8503, Kanagawa, Japan

**Keywords:** femtosecond laser ablation, direct energy deposition, radiative cooling, anti-reflective surfaces

## Abstract

The reflectance (*R*) of linear and circular micro-gratings on c-plane sapphire Al_2_O_3_ ablated by a femtosecond (fs) laser were spectrally characterised for thermal emission ∝(1−R) in the mid-to-far infrared (IR) spectral range. An IR camera was used to determine the blackbody radiation temperature from laser-patterned regions, which showed (3–6)% larger emissivity dependent on the grating pattern. The azimuthal emission curve closely followed the Lambertian angular profile $\propto \cos\theta_{a}$ at the 7.5–13 μm emission band. The back-side ablation method on transparent substrates was employed to prevent debris formation during energy deposition as it applies a forward pressure of >0.3 GPa to the debris and molten skin layer. The back-side ablation maximises energy deposition at the exit interface where the transition occurs from the high-to-low refractive index. Phononic absorption in the Reststrahlen region 20–30 μm can be tailored with the fs laser inscription of sensor structures/gratings.

## 1. Introduction

Fs laser machining has been a versatile technique for fabricating micro- to nano-scale patterned surfaces for precise tailoring of optical [1,2,3,4,5], thermal [6,7], tribological [8,9,10,11], and wettability [12,13,14,15] properties of different materials’ surfaces from reflective metals [16,17,18] to transparent dielectrics [19,20,21]. Fs laser fabrication via direct laser writing enables the realisation of metasurfaces—defined as sub-wavelength structures—with featured sizes on the scale of a few micrometers [17]. This enables a simple prototyping capability for fabricating 2D and 3D patterns, making modifications on mid-to-far IR spectrum and extending even further into the THz spectral range (millimetre scale) [22]. In this range, standard projection lithography approaches are feasible in terms of resolution but they are not highly demanding. However, high-aspect-ratio fabrications become demanding when the depth approaches the wavelength scale (sub-millimetre), e.g., optical vortex generators [23]. Fs laser machining is capable of producing high-aspect-ratio fabrications with high precision on dielectrics and metals [16,17,18,19,20,21]. The fabrication of subwavelength structures with controlled periodicity and depth enables potential applications in infrared sensing, radiative cooling, and optics. Representative studies include 2D laser-induced periodic surface structures (LIPSSs) on SiC for enhanced solar absorptance [24], nanotexturing on Mo for enhanced and selective absorption [25], surface treatment of ceramics for high-temperature solar absorbers [26,27,28], as well as micromilling on Si [29].

The reflectivity of a surface must be controlled for its anti-reflective and highly emissive properties, which can be tailored by fs laser texturing at IR wavelengths [30,31]. Indeed, the reflectance $R_{0}$ of a mirror-finished surface will change to $R=R_{0}\exp\left[-16\pi^{2}\delta^{2}/\lambda^{2}\right]$ due to surface roughness δ (RMS) [32]. The decrease in *R* contributes to the increase in absorbance *A*, which in turn enhances the emissivity *E*. Therefore, the phenomenon of *R* reduction has a direct link to radiative cooling applications, which has been demonstrated in previous studies [33,34,35,36]. The mid-to-far IR spectral window at the Reststrahlen band, defined between the transverse optical (TO) and longitudinal optical (LO) phonons with energies $\hbar \omega_{TO}$ and $\hbar \omega_{LO}$, in the energy dispersion ω(k) (where wavevector k=2π/λ) can be explored for sensor and slow light applications. This is because both real and imaginary parts of the refractive index are very large ($\tilde{n}=n+i\kappa$ attaining n≈κ≈15), especially the $\tilde{n}$ found in polar materials. In the spectral regions where n≈κ, a strong (even near to perfect) absorption condition can be achieved similarly to the strong absorption observed in laser-driven breakdown plasmas. Indeed, when the real part of the permittivity ($n^{2}-\kappa^{2})$ is approaching 0 (where permittivity $\varepsilon =n^{2}-\kappa^{2}+i2n\kappa$), the epsilon-near-zero (ENZ) conditions can be realised, which occur in perfect absorbers and near-breakdown plasmas (the real part of permittivity Re(ε)=0 is the breakdown definition). In Al_2_O_3_, ellipsometry measurements at this spectral region were shown to be suitable for determining the permittivity $\varepsilon ={\tilde{n}}^{2}$ at different surface orientations [37]. The static and high-frequency dielectric constants for the electric E-field perpendicular to the c-axis of Al_2_O_3_ (c-plane used in our study) are $\varepsilon_{0}=3.1$ and $\varepsilon_{\infty }=9.4$, respectively, and they are related to each other via the Lyddane–Sachs–Teller rule $\frac{\varepsilon_{0}}{\varepsilon_{\infty }}=\frac{\omega_{LO}^{2}}{\omega_{TO}^{2}}$. The complex dielectric function (permittivity) at a wavelength of λ (or cyclic frequency ω=2πc/λ) comprises contributions from both phonons and electrons, as described by the Drude model. These contributions correspond to the IR region (Reststrahlen band) and the optical spectral regions, respectively. The complex dielectric function is given by [32]:

(1) $$\varepsilon \left(\omega \right)=\varepsilon_{\infty }\left[1+\frac{\omega_{LO}^{2}-\omega_{TO}^{2}}{\omega_{TO}^{2}-\omega +i\omega \Gamma }-\frac{\omega_{p}^{2}}{\omega (\omega -i\gamma_{fc})}\right],$$

where the electron plasma frequency $\omega_{p}=\sqrt{\frac{4\pi N_{e}e^{2}}{\varepsilon_{\infty }m^{*}}}$ is defined by electron density $N_{e}$, electron charge *e*, and its effective mass $m^{*}$; $\gamma_{fc}=\frac{e}{m^{*}\mu }$ is the free-carrier damping constant determined by the carrier (electron) mobility μ, and Γ is the phonon damping constant. The Reststrahlen bands possess particularly strong absorption resonances (e.g., at the TO band), where the Kramers–Kronig relations dictate that the real permittivity becomes negative over a narrow region. This results in strong phonon-photon interactions, leading to the formation of surface phonon polaritons (SPhPs). The dispersion relation at this region has a typical anti-crossing signature [38]:

(2) $$\omega^{2}\left(k\right)=\frac{1}{2\varepsilon_{\infty }}\left[c^{2}k^{2}+\varepsilon_{\infty }\omega_{LO}^{2}\pm \sqrt{{\left(c^{2}k^{2}+\varepsilon_{\infty }\omega_{LO}^{2}\right)}^{2}-4\varepsilon_{\infty }c^{2}k^{2}\omega_{TO}^{2}}\right].$$

where ω is the angular frequency as a function of wavevector *k*, and *c* is the speed of light in the material. One of c-plane sapphire’s pronounced Reststrahlen bands is at the TO_2_-LO_2_ pair 439–481 cm^−1^ [37,39]. In this band, strong reflectivity occurs and was present in our experiments. Equation (2) is useful to define the angle of incidence $\theta_{in}$ at which the SPhP can be launched using IR photons. This occurs at the wavevector (*k*)-matching condition facilitated by an optical element, e.g., a grating with period Λ or a prism. The phase matching conditions for IR photon, SPhP surface wave, and surface grating with period Λ are:

(3) $$\frac{2\pi }{\lambda_{IR}}\sin\theta_{in}=\frac{2\pi }{\lambda_{SPhP}}+m\frac{2\pi }{\Lambda },$$

where *m* is an integer. The phase (momentum)-matching conditions Equation (3) are also applicable for the extraction of IR (black body) radiation from the TO phonon modes parallel to the surface $k_{\|}=2\pi /\lambda_{SPhP}$ using the same grating. This was demonstrated on the SiC surface with a grating of Λ≈11.36 μm, which corresponded to the Λ=0.55λ conditions and out-coupling angle $\theta_{out}$ instead of $\theta_{in}$ in Equation (3) [40]. Moreover, since the grating was sub-wavelength relative to the emitted IR photons, the emissivity exhibited strong directionality due to coherence, a phenomenon known as Wolf’s effect [41,42]. This effect can be used to enhance IR blackbody emission and holds potential for applications in radiative cooling [40]. The Wolf’s effect is influenced by the depth of the gratings, and the use of circular gratings to facilitate the experimental observation of out-coupled IR light was a key motivation for this study. It was found that the reflectivity increases at 385–388 cm^−1^ (TO_1_ mode) and decreases at 633 cm^−1^, while the TO_4_ modes were smoothed in the spectral reflectivity (*R*) profile following fs laser ablation at 1030 nm/280 fs and surface patterning with ablation ripples [43].

As a crystal form of Al_2_O_3_, sapphire has extraordinary thermal, optical, and mechanical properties. Its physical properties, such as high thermal conductivity, resistance to thermal stress, low thermal expansion and high hardness, make it adaptable under extreme conditions [44]. However, sapphire is less compatible with conventional photo-assisted machining techniques due to its wide bandgap (>8 eV). An fs laser is a suitable tool to pattern sapphire with precise structures [45], such as grating to excite Fano resonances; thus, the optical response in the Reststrahlen band can be further enhanced [46,47]. For instance, in periodically modulated dielectric films, Fano-like coupling with unbound planar waveguiding modes enables precise control over optical properties and resonance features [48,49].

Here, we demonstrated the depth control in fs laser micro-machining of a c-plane sapphire Al_2_O_3_ to engineer reflectivity bands for enhanced emission in mid-to-far IR and radiative cooling. The depth and aspect ratio control of surface texturing reduced the reflectance, *R*, which corresponded to an enhanced absorbance, *A* which is directly related to the emittance, *E*. The emittance and use of Wolf’s effect on the IR material enables controlled extraction of radiation tailored to specific IR bands.

## 2. Materials and Methods

### 2.1. Ablation of Grooves on Sapphire with a Gaussian Beam

**Figure 1:**
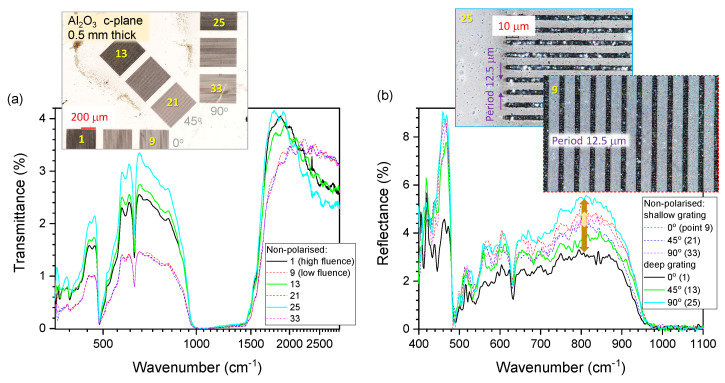
Front-side ablation of gratings on Al_2_O_3_ with spectra measured using synchrotron radiation. (**a**) Transmittance *T* (wavenumber is in log-scale) and (**b**) reflectance *R* measured in mid-to-far IR spectral range; insets show optical images of laser ablated Al_2_O_3_ at different orientations. Single-point measurements were carried out on the laser-ablated regions (numbers are indicated on insets); the fabrication was in CD mode explained in Section 2.1 with the laser setup: central wavelength λ=1030 nm, pulse duration $t_{p}=230$ fs, scanning speed $v_{s}=10$ mm/s, constant density $D_{SG}=100$ mm^−1^ for shallow gratings, and $D_{DG}=500$ mm^−1^ for deep gratings. The effective pulse repetition rate for shallow gratings was 1 kHz and for deep gratings was 5 kHz; the pulse energy was $E_{p}=10$ μJ, and numerical aperture was NA=0.45 (focal diameter 1.22λ/NA≈2.8 μm); the grating period was 12.5 μm with *h* = 1–10 μm depth of the ablated-groove; depth defined by ablation fluence and number of repeated passes $N_{p}=1,2,3$ (without change of vertical focal spot position).

A Pharos fs laser system with a pulse duration $t_{p}$ = 230 fs was used for the machining of 1.5×1.5 cm^2^ sapphire chips. The laser system has Aerotech’s Position-Sensitive Output (PSO)/constant density (CD) mode, where the pulse placement is effectively independent (to an extent) of laser frequency, scanning speed, and scanning accelerations. The laser pulse was shot when the cumulative travel distance of all 3 stages reached a predetermined distance 1/D, where D is pulse density. The CD mode is advantageous over the constant frequency mode when precise laser pulse placement with constant pulse-to-pulse spacing is required. The fundamental wavelength λ=1030 nm was used for front-side ablation in CD mode with $D_{SG}=100$ mm^−1^ for shallow gratings and $D_{DG}=500$ mm^−1^ for deep gratings as Figure 1. The effective repetition rate $f_{eff}$ was adjusted by the laser system to meet the density requirement as $f_{eff}=Dv_{s}$, where scanning speed $v_{s}=10$ mm/s ($f_{SG}=1$ kHz for shadow gratings and $f_{DG}=5$ kHz for deep gratings). The fabrication parameters are pulse energy $E_{p}=10$ μJ, numerical aperture NA=0.45 (focal diameter 1.22λ/NA≈2.8 μm), the grating period Λ = 12.5 μm, and the depth of the ablated-groove *h* = 1–10 μm. The depth was defined by ablation fluence and the number of repeated passes $N_{p}=1,2,3$ (without change of vertical focal spot position). The experimental parameters are also detailed in the caption of Figure 1.

**Figure 2:**
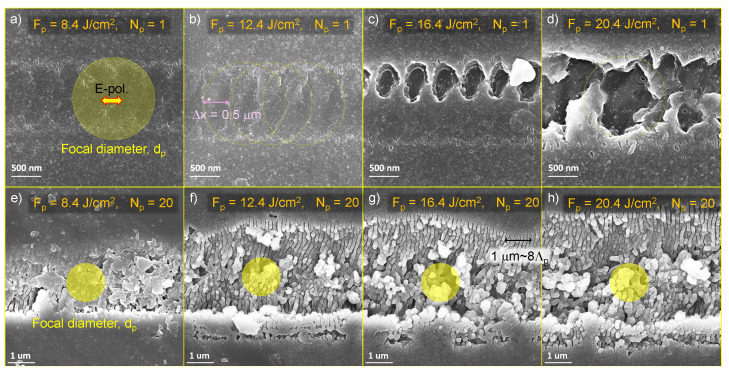
Back-side ablation of Al_2_O_3_. SEM images of grooves on sapphire ablated with different pulse fluence $F_{p}$ and number of passes $N_{p}$; $F_{p}$ increased from the left $8.4\;{J/\mathrm{cm}}^{2}$ to the right $20.4\;{J/\mathrm{cm}}^{2}$. (**a**–**d**) Single-pass ablation. (**e**–**h**) Twenty-pass ablation. Focusing with the NA=0.45 objective lens, the focal spot $d_{p}\approx 1.22\lambda /NA=1.4$ μm; a 515 nm/230 fs Pharos laser was used in CD mode with a pulse density of 2000 mm^−1^ and a scanning speed of $v_{s}$ = 15 mm/s, an effective repetition rate of $f_{eff}=Dv_{s}=30$ kHz, and a number of pulses per focal diameter of $d_{p}$ is $N=d_{p}/\Delta x\approx 3$. Linear polarisation of the writing beam was aligned with the scan direction (horizontal).

**Figure 3:**
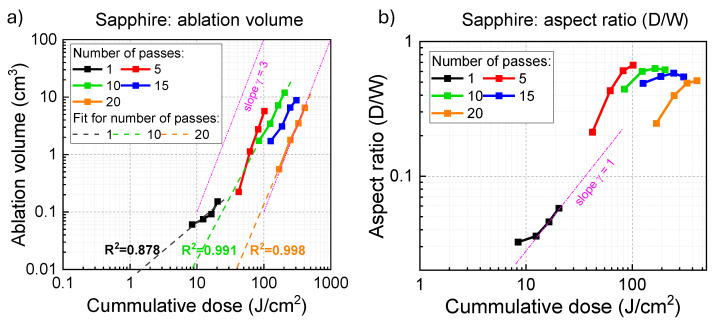
Back-side ablation of Al_2_O_3_. (**a**) The effective ablation volume and (**b**) aspect ratio of ablated grooves on sapphire engraved by a 515 nm/230 fs Pharos laser in CD mode. The x-axis is the total dose $F_{p}\times N_{p}$, where $N_{p}$ is the number of passes. The pulse-to-pulse separation was Δx=0.5 μm for the constant pulse density mode of 2000 mm^−1^ corresponding to N≈3, laser repetition rate. The pulse fluence $F_{p}=E_{p}/\left(\pi {\left[d_{p}/2\right]}^{2}\right)$ ($E_{p}$ pulse energy), the ablation volume (conical pyramid) $V=\pi {\left(W/2\right)}^{2}D/3$ and the aspect ratio α=D/W were calculated with *W* the width and *D* the depth of the groove; R^2^ is the coefficient of determination for each linear fitting. Focusing with the NA=0.45 objective lens, $d_{p}=1.22\lambda /NA=1.4$ μm.

The remaining fabrications were back-side ablation in CD mode with a density D=2000 mm^−1^. The scanning speeding was $v_{s}$ = 15 mm/s, and the effective repetition rate can be calculated as $f_{eff}=Dv_{s}=30$ kHz. These fabrications used the laser beam with the central wavelength λ = 515 nm, pulse energy up to EP = 12.5 μJ (power P=2.5 W), and a numerical aperture NA=0.45 (with a focal spot of 1.22λ/NA ≊ 1.4 μm). The number of pulses per focal spot $N=d_{p}/\Delta x\approx 3$ at the constant pulse density mode of 2000 mm^−1^ (the pulse-to-pulse spacing Δx=0.5 μm). The laser was focused on the back side of the sapphire chips to reduce the influence of ablation debris and to localise the intensity at the interface between the exit sapphire surface and air. Compared with front-side ablation, the back-side ablation tends to accumulate less ablation debris which are obstructions of laser ablation (Supplementary A in Ref. [50]), and its efficiency is one or even two magnitudes higher than the efficiency of front-side ablation [51]. The machining was achieved under the condition a of varying fluence per pulse $F_{p}=8.4,\;12.4,\;16.4,\;20.4$ J/cm^2^ and a different number of passes $N_{p}=1,\;5,\;10,\;15,\;20$. For each condition, 4 grooves were engraved to calculate the average dimension parameters (width, depth, ablation volume, and aspect ratio) for a more representative result. The results obtained under this setting are presented in Figure 2 and Figure 3.

**Figure 4:**
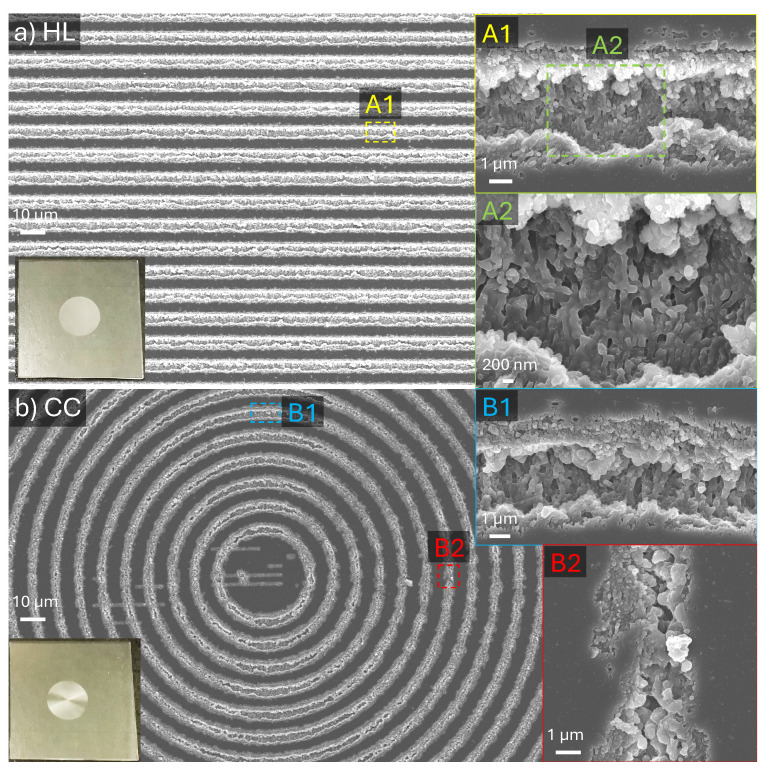
Back-side ablation of Al_2_O_3_. SEM images of large-area pattern fabrication with a diameter of 0.5 cm on 1.5×1.5 cm^2^ sapphire chips ablated by a 515 nm/230 fs Pharos laser in CD mode, pulse fluence $F_{p}$ = $20.4\;{J/\mathrm{cm}}^{2}$ (pulse average intensity $I_{p}=F_{p}/t_{p}=88.7$ TW/cm^2^), number of passes $N_{p}$ = 10. (**a**) The fabricated area is filled with horizontal hatching (HL). (**b**) The same fabrication condition for the concentric circle (CC) pattern; both HL and CC patterns have the grating period Λ=10 μm; the insets (left-bottom) are optical pictures of the fabricated sapphire chips; the insets A1, A2, B1, and B2 display enlarged SEM images corresponding to the labelled areas.

**Figure 5:**
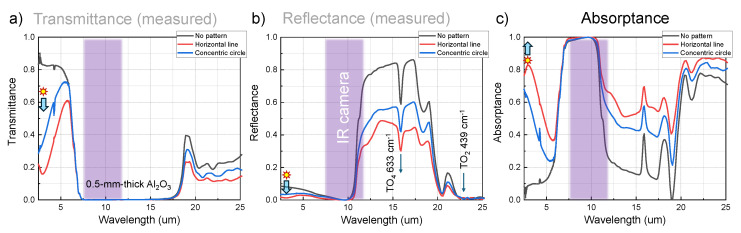
Laboratory-based FTIR spectra of the horizontal line (HL) and concentric circular (CC) grating structures (Figure 4), collected using a Bruker Vertex spectrometer with an internal Globar™ IR source (unpolarised). Experimentally measured reflectance *R* (normalized to Au mirror R=1) and transmittance *T*. Then the absorptance *A* is calculated from the energy conservation A+R+T=1. Changes of R,T (measured), and *A* (calculated) at 7.5–13 μm caused emittance changes when imaged by IR camera (Figure 6); highlighted by a rectangular block. A star marker with an arrow shows a tendency of increased *A* at ∼4.3 μm as compared with unstructured Al_2_O_3_.

**Figure 6:**
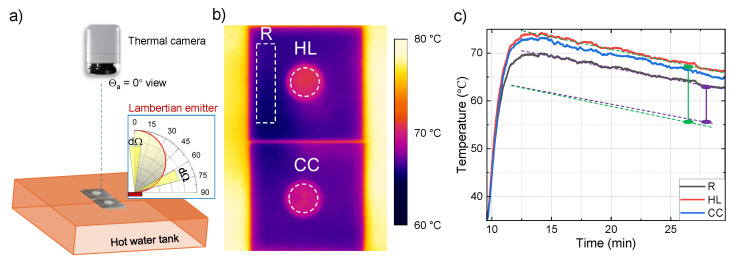
(**a**) Temperature measurement setup: sapphire samples were placed in the centre of a hot water tank (polypropylene), with a thermal camera (Optris PI 160, Optris GmbH, Berlin, Germany) positioned above (azimuth $\theta_{a}=0^{{}^{\circ}}$) the sample to capture temperature data. Inset shows an emissivity angular diagram for the Lambertian source $I_{0}\cos\theta_{a}$, dΩ is the solid angle wedge into which the black body source emits (see discussion in the text). (**b**) Thermal image captured by the camera, showing monitored areas with the horizontal line (HL) and concentric circle (CC) patterns, as well as a non-patterned sapphire surface as a reference (R). (**c**) Cooling transients: temperature change from the monitored areas.

**Figure 7:**
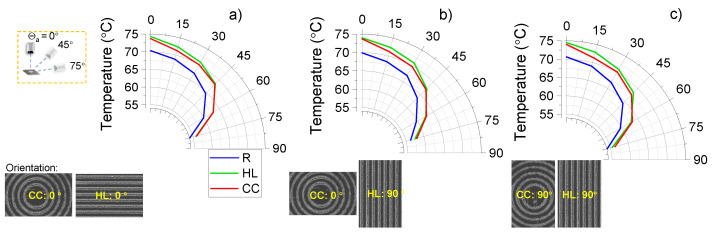
Lambertian-like emitter surfaces. Measurement of temperature of sapphire samples at the azimuthal observation angles from $\theta_{a}=0^{{}^{\circ}}$ to $75^{{}^{\circ}}$ angle at $\phi =0^{{}^{\circ}}$ and $90^{{}^{\circ}}$ orientations (orientation in the plane of the sample). (**a**) HL at $0^{{}^{\circ}}$ and CC at $0^{{}^{\circ}}$ orientation. (**b**) HL at $90^{{}^{\circ}}$ and CC at $0^{{}^{\circ}}$ orientation. (**c**) HL at $90^{{}^{\circ}}$ and CC at $90^{{}^{\circ}}$ orientation. The setup is the same as Figure 6 to record the temperature at different angles like the insert shown in (**a**).

**Figure A1:**
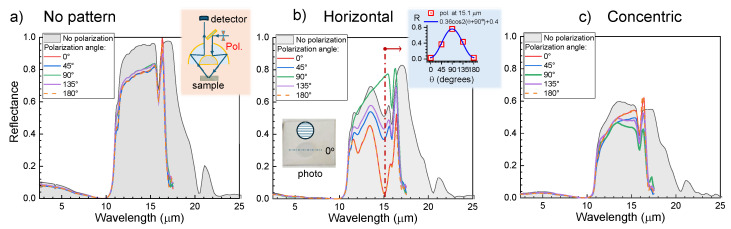
Reflectance FTIR spectra R(λ) from c-plane Al_2_O_3_ without laser patterned gratings as well as from 0.5 to mm-diameter Λ=10 μm period linear and circular gratings in (**a**–**c**), respectively, at different polarisation angles θ; inset in (**a**) shows the geometry of measurements. The top inset in (**b**) shows the angular dependence of *R* vs. θ angle of linearly polarised incident IR radiation from a thermal IR source (Globar™) at λ=15.1 μm. Numerical aperture of Cassegranian objective lens NA=0.5, which defined focal diameter $d_{s}=1.22\lambda /NA\approx 45.8$ μm at λ=15 μm, i.e., $d_{s}\approx 4\lambda$. Note: *R* spectra are shown only up to a wavelength of 17.5 μm, as the mesh polariser introduces significant noise beyond this range, making the spectrum unreliable due to its inherent design limitations.

After optimising the fabrication conditions, two distinct patterns were produced over a large area, both with a grating period of Λ=10 μm and a patterned area diameter of 5 mm. The first pattern consists of gratings shaped as concentric circles (CC), while the second features evenly spaced horizontal lines (HL) arranged within a circular outline. Both patterns were back-side ablated using the wavelength λ=515 nm under identical scanning and focusing conditions (see parameter set in the caption of Figure 4) and were subsequently used for the measurement of infrared spectra (Figure 5 and Figure A1) and emissivity (Figure 6 and Figure 7).

### 2.2. Structural Characterisation

To characterise the dimensions of the machined sapphire grooves, a 3D profile was captured using a Bruker Contour GT-K 3D Optical Profiler (Bruker Nano Surfaces, Billerica, MA, USA) at a magnification of 115×. Using the open-source software, Gwyddion (Version 2.67), the average depth and width of grooves were calculated. The depth (*D*) was defined as the distance from the bottom of the groove to the surface plane, and the width (*W*) was defined as the distance between two edges of the groove. The aspect ratio was defined as the ratio of the previous two quantities (α=D/W) and the ablation volume was defined as a conical pyramid ($V=\pi {\left(W/2\right)}^{2}D/3$). The results are presented in Figure 3. The surface morphology was captured using a scanning electron microscopy (SEM). The RAITH 150TWO electron beam lithography writer (Raith GmbH, Dortmund, Germany) was used in the in field emission mode using back-scattered electrons. The sapphire samples were coated with 20 nm Cr using a Quorum Q150T ES Plus Spin Coater (Quorum Technologies Ltd., Laughton, UK) before SEM imaging.

### 2.3. Spectral Characterisation

Laboratory-based Fourier transform infrared (FTIR) spectra were measured in both reflection and transmission modes at 2.5–25 μm using a Bruker Vertex 70 spectrometer coupled with a Hyperion 1000/2000 FTIR microscope (Bruker Optik GmbH, Ettlingen, Germany). The absorption was then calculated according to Kirchhoff’s law where A=1−T−R (scattering is accounted for by reflection and absorption). The measured transmittance, reflectance, and calculated absorptance were analysed as Figure 5.

Synchrotron-based FTIR spectra were also acquired at the Infrared Microspectroscopy (IRM) beamline, Australian Synchrotron (Clayton, VIC, Australia), using a Bruker Vertex 80v spectrometer coupled with a Hyperion 3000 FTIR microscope (Bruker Optik GmbH, Ettlingen, Germany) and a helium-cooled Si:B photodetector. The focal spot was ∼30 μm; a 20^×^ objective lens (NA=0.6) and an identical pair of 36^×^ objective lens (NA=0.5) were used for reflection and transmission measurements, respectively. All the synchrotron–FTIR spectra were recorded within a spectral range of 4000–340 cm^−1^ using 4 cm^−1^ spectral resolution. Blackman-Harris 3-Term apodisation, Mertz phase correction, and a zero-filling factor of 2 were set as default acquisition parameters using the OPUS 8.0 software suite. A non-polarised synchrotron-IR beam was used. The results are presented in Figure 1 with the fabrication conditions specified in the figure caption.

### 2.4. Thermal Emission

The temperature profiles of the sapphire samples were captured using a thermal imaging camera (Optris PI 160, Optris GmbH, Berlin, Germany) which operates in the 7.5–13 μm IR band. When the temperature of a hot water tank (made of polypropylene with a water volume of 15.5×9.5×6.0=883.5 cm^3^) becomes stable, the sapphire samples were placed in the centre of the water tank to be heated up. As the hot water tank progressively cooled in the ambient room environment, the sapphire samples were cooled as well. The thermal camera was placed directly above the sapphire samples to capture the temperature variations during the heating and cooling phases. A thermal monitoring software (Optris PIX Connect Version 3.23, Optris GmbH, Berlin, Germany) was used to connect the camera to the computer and was used to monitor the labelled sample areas.

## 3. Results and Discussion

### 3.1. Effect of Orientation and Depth of Ablated Groves on *T* and *R*

Determining emittance as absorbance (E≡A) requires direct measurements of transmittance *T* and reflectance *R* (A=1−R−T). Measurements were carried out on laser-ablated gratings with different orientations and depths using the synchrotron radiation (IR beamline at Australian Synchrotron) as shown in Figure 1. To maximise *A*, *R* should be minimised. Therefore, to reduce the reflectivity of the material’s surface with refractive index $n_{s}$ (for light incidence from the air with $n_{air}=1$), a periodic square pattern of sub-wavelength structures with period *p* and depth *h* is typically employed [52]. The sub-wavelength pattern should satisfy $\frac{p}{\lambda }<\frac{1}{n_{air}\sin\theta_{i}+n_{s}}$ [53,54] with the height $h=\frac{\lambda }{4n_{eff}}$ (the thinnest layer) of the pattern; *p* is the pattern’s period; $\theta_{i}$ is the angle of incidence onto the front surface from air-to-sample. The reflection is minimised when the refractive index of the anti-reflection coating is the geometric mean of the refractive index on either side of the material $n_{eff}=\sqrt{n_{s}n_{air}}$. For the laser-patterned surface, the volume fraction of material *f* and air (1−f) defines the effective refractive index $n_{eff}^{2}=\left(1-f\right)n_{air}^{2}+fn_{s}^{2}$. This shows how period and depth are both important for the reduction of *R* (for intensity $I=E^{2}$) [55]:

(4) $$R\equiv |r^{2}|=\frac{r_{1}^{2}+r_{2}^{2}+2r_{1}r_{2}\cos2\delta }{1+r_{1}^{2}r_{2}^{2}+2r_{1}r_{2}\cos2\delta },$$

where reflections from two interfaces are $r_{1}=\frac{n_{air}-n_{eff}}{n_{air}+n_{eff}}$ and $r_{2}=\frac{n_{eff}-n_{s}}{n_{eff}+n_{s}}$ (for *E*-field), respectively, $n_{s}$ is the refractive index of the substrate/sample, and the phase delay term $\delta =\frac{2\pi }{\lambda }n_{eff}h$ for the *h* thickness of the anti-reflection layer.

Figure 1 shows measured *R* and *T* spectra at mid-far-IR for gratings with fs laser ablation on the c-plane of Al_2_O_3_ at different orientations and depths h≈1,5,10 μm. The dichroic ratio for reflectance parallel and perpendicular to the ablated groves $d_{R}=R_{\|}/R_{⊥}$ was largest ∼2% for the deepest grating at $\tilde{\nu }=800$ cm^−1^ (λ=12.5 μm) which corresponds to the period of the grating.

Strong *R* modulation ∼9% is observed at the Reststrahlen band TO_2_-LO_2_ pair 439–481 cm^−1^ (22.8–20.8 μm) and was strongest for the deepest pattern. Strong dichroism in *R* and *A* can be explored for sensor applications of surface-enhanced IR absorption (SEIRAS), which is widely used at shorter IR wavelengths. The benefit of the Reststrahlen region is the possibility to launch SPhP (surface phonon polariton) at the TO-band using a grating of Λ ≤ λ (see dispersion Equation (2)). In this regime, slow light at large-*k* is realised with the local electric field enhancements $E_{loc}\propto \varepsilon E_{0}∼n^{2}E_{0}$ ($E_{0}$ is the incident field), where intensity follows $I=E^{2}\propto n^{4}$ with n≈ 10–20. These enhancements can become significantly stronger similar to those observed in the visible spectral range. Polarisation dependence on reflectance *R* (Figure A1) shows very strong modulation ΔR≈0.8 for incidence along and across the linear grating pattern. The grating acts as an optical element to add 2π/Λ momentum to launch SPhP (Equation (3)) or to extract IR emission via Wolf’s effect.

### 3.2. Characterisation of Back-Side Ablation: Threshold, Structure, *R*, and *T* Spectra

Figure 2a–d shows SEM images of $N_{p}=1$ pass laser ablation on sapphire with different fluences 8.4, 12.4, 16.4, 20.4 ${J/\mathrm{cm}}^{2}$ at the condition N≈3 pulse overlap per focal diameter. When the fluence was below 12.4 ${J/\mathrm{cm}}^{2}$, the surface had structural modifications, however, without clear ablation pits/grooves. At a fluence of 16.4 ${J/\mathrm{cm}}^{2}$, clear ablation structures appear on sapphire. By increasing the pulse fluence to 20.4 ${J/\mathrm{cm}}^{2}$, material removal at ablation sites becomes deeper, forming a continuous trench. At the same fluence, for $N_{p}=20$ passes, sapphire was ablated with debris coming out of the surface even at the lowest pulse fluence (Figure 2e–h). This signifies the presence of a cumulative effect in sapphire ablation. By measuring the ablation depth and width, and by calculating the ablation volume versus the cumulative dose, the ablation threshold at different conditions can be estimated as shown in Figure 3a. Sapphire’s ablation threshold is around 1.6 ${J/\mathrm{cm}}^{2}$ for an $N_{p}=1$ pass. It is close to a single pulse ablation threshold of sapphire ∼2 J/cm^2^ considering N=3 pulse accumulation over the focal spot at the used writing conditions.

For the back-side ablation, ripples with a period of Λ=145±10 nm were observed (Figure 2g). This period closely matches the expected value for internal ripples, calculated as [λ/n]/2=151 nm, where the laser wavelength is λ=515 nm and the refractive index of sapphire is n≈1.77. The observed pattern is typical for normal ripples with grating-like structures perpendicular to the linear polarisation of the electric *E* field. Interestingly, surface ripples for the front-side (most usual experimental case) ablation appear once multi-pulse irradiation takes place starting from two pulses overlapped N=2. For the back-side ablation, ripples were prominent only when larger pulse fluence was used, and ablation was larger than the geometrical focal spot and evolved on the side walls of ablation grooves. One possible explanation for the absence of ripples close to the threshold of their formation is photon pressure. Indeed, the average intensity $I_{p}=10$ TW/cm^2^ (close to the ablation threshold) generates forward pressure along the beam $I_{p}/c\approx 0.33$ GPa, which facilitates forward ablation of the skin-depth of highly excited and absorbing sapphire at the back-side focus. Moreover, the reflection of the incoming laser beam from the skin region ionised Al_2_O_3_ deposits twice the photon momentum (doubles the pressure). The obvious shallow removal of material over exactly the focal spot was discernible by SEM.

The ablated volume follows the γ=3 power law on the dependence of fluence/dose $Volume\propto Dose^{\gamma }$ (Figure 3). This is consistent with two-photon absorption (TPA) in energy deposition. It is noteworthy that even for a single pass ablation, it was three pulses overlapping per single focal diameter. Moreover, a single pulse was up to ten times more intense as compared with the ablation threshold. This resulted in a strongly damaged Al_2_O_3_ and power scaling of ablation, which is different as compared with pristine alumina [56]. The density of deposited energy depends on the pulse fluence and skin depth $w_{ab}=2AF_{p}/l_{skin}\propto \frac{n_{e}}{n_{cr}}F_{p}$, where skin depth $l_{skin}=1/\alpha ={\left(4\pi \kappa /\lambda \right)}^{-1}$, $n_{e}$ is electron density, $n_{cr}$ is the density of critical plasma at the wavelength of irradiation, $F_{p}$ is the pulse fluence and *A* is the absorbance. When electrons are generated by TPA, $w_{ab}\propto F_{p}^{3}/n_{cr}$, the scaling is confirmed in experiments. Once the density of deposited energy $w_{ab}$ (per volume) exceeds the binding and ionisation energy of the material (Al_2_O_3_), the ablation starts [57].

The ionisation mechanism is usually discussed in terms of the adiabaticity or Keldysh parameter $\gamma_{K}$ (see Appendix B). The multiphoton ionisation has a quantum tunnelling character when $\gamma_{K}\ll 1$, and classical multi-photon ionisation when $\gamma_{K}\gg 1$. For the pulse fluence $F_{p}=20$ J/cm^2^ (Figure 3), the peak intensity $I_{max}$ can be estimated as twice the average pulse irradiance/intensity $I_{max}=2I_{p}\approx$ 177 TW/cm^2^, which defines $\gamma_{K}=0.96$. If fluence $F_{p}$ was relative low at 10 J/cm^2^, $I_{max}=2I_{p}\approx$ 89 TW/cm^2^, $\gamma_{K}=1.36$. For the used range of intensities of ablation and laser machining (Figure 3), the absorption/ionisation had a mixed muti-photon and tunelling character since the conditions $\gamma_{K}\ll 1;\gamma_{K}\gg 1$ were not fulfilled. These nonlinear ionisation mechanisms were providing seeding electrons for the avalanche ionisation (inverse bremsstrahlung), which is more efficient for longer wavelengths due to free carrier absorption scaling as $\lambda^{2}$.

The removal of skin depth layers by ablation can explain the experimental data (Figure 3a). Figure 3b shows the aspect ratio increases with the increase of cumulative deposited dose on the sapphire’s surface. The aspect ratio was increasing linearly with the increase of dose/fluence near the ablation threshold. At repeated scans with a larger cumulative dose, the depth was increasing faster than the width. This is consistent with the Gaussian intensity profile, which decays laterally as $1/e^{2}$ while axially (along propagation) as 1/e. The depth increase saturates after several scans since the focal spot position was not changed with respect to the ablated depth in consecutive scans.

Sapphire has a higher ablation threshold due to its wide bandgap (>8 eV) compared with other dielectric materials such as SiC (3 eV), making it more difficult to form periodic structures [58]. In contrast, SiC exhibits better control over surface periodicity due to its lower ablation threshold [24]. However, sapphire excels in thermal stability (>2000 °C vs. SiC’s 1600 °C decomposition temperature), lower thermal effects (lower heat diffusion than metals and ceramics), and hardness (second only to diamond). These properties make sapphire preferable for specific radiative cooling scenarios, such as high-temperature and high-pressure applications.

The large areas were fabricated using the 20.4 ${J/\mathrm{cm}}^{2}$ Gaussian irradiation with $N_{p}=10$ passes on the back side of sapphire, with 0.1 μm depth offset per pass during scanning. Figure 4 shows SEM images of two patterns at different magnifications, with detailed micro-structure of the groove (A1, B1, B2) and nano-scale ripples (A2). The microstructure is polarisation-dependent, where ripples were more pronounced when polarisation was perpendicular to the scanning direction. When the laser was scanning along the horizontal direction, and polarisation was vertical, deeper grooves and ripples were formed at the bottom. Laser-ablated patterns affected spectral properties. The HL pattern shows both lower reflectance and transmittance at mid-IR as compared with the CC pattern, leading to higher absorptance as Figure 5. The possible reason is that the HL pattern is deeper. Additionally, more ripple formation in the HL pattern due to the polarisation of fs laser irradiance largely enhanced surface roughness, promoting broadband absorption in the IR range. This is because fs laser-induced nanostructures amplify absorption by acting as micro-resonators for IR emission [59]. The absorptance at 7–11 μm is near to 1 due to a large 0.5 mm thickness and the strong absorbance of sapphire [60]. Moreover, absorption at 11–19 μm was increased from 0.2 to 0.6 with the HL patterning. The emissivity enhancement observed in the better-designed structure is more pronounced compared to the fabrication shown in Figure 1. The spectra in Figure 1 were obtained using unpolarised synchrotron radiation, as detailed in Section 2.3. Apart from that, the fabricated structures differ in depth, width, and presence of ripple formation due to the difference in ablation parameters. These differences, along with the influence of IR polarisation conditions, contribute to the discrepancies observed in the FTIR spectra between Figure 1 and Figure 5.

### 3.3. Thermal Emission and Angular Dependence

According to the Stefan–Boltzmann law $P=\sigma AT^{4}$, the total power radiated by a black body *P* [W] is proportional to the $T^{4}$, where $\sigma =\frac{2\pi^{5}k_{B}^{4}}{15h^{3}c^{2}}=5.67\times 10^{-8}$ W/m^2^K^4^ is the Stefan–Boltzmann constant defined by the basic constants ($k_{B}$ Boltzman’s, *h* Planck’s, and speed of light *c*), *A* [m^2^] is the surface area, and *T* [K] is the temperature of the black body [61].

The observed radiant intensity *I* [W/srad], i.e., the radiant flux (or irradiance [W/m^2^]) per unit of solid angle, from an ideal diffusely emitting (or reflecting) surface is directly proportional to the cosine of the angle $\theta_{a}$ between the observer’s line of sight and the surface normal. Hence, for the ideal emitter (reflector) $I=I_{0}\cos\theta$, the number of photons per second (J/s = W) emitted into the wedge dΩ at angle $\theta_{a}$ is $I\cos\theta_{a}d\Omega dA$, where dΩ is the solid angle to which the emitting area dA subtends at observer (e.g., detector). Therefore, the emission rate in photons/s in a normal and off-normal direction is proportional to the area of the wedge $\cos\theta_{a}d\Omega$. However, for a Lambertian emitter, the intensity in photons/(s·sr·m^2^) remains the same in both the normal and off-normal directions. This is because the emittance scales with $\cos\theta_{a}$, and the solid angle variation compensates for the intensity change. Since the blackbody radiation is uniform with no directionality, the radiation intensity *L* of a blackbody depends on the radiant emittance $E_{T}=\sigma T^{4}$ as $L=E_{T}/\pi$ [W/(m^2^srad)], where *L* is blackbody radiation intensity and unit is power per unit area per unit solid angle; σ is Stefan–Boltzmann constant.

Next, surface emissivity with laser-ablated patterns at different orientations was tested to check if such patterned surfaces have a Lambertian-emitter nature or if there is Wolf’s effect with preferred directions of light extraction (Equation (3)). An IR camera integrating response over 7.5–13 μm spectral window was used. Notably, measurements of surface temperature (black body radiation) at different azimuthal tilt $\theta_{a}$ from the ideal Lambertian emitter will result in different surface temperature readings due to different photon numbers per time (power) emitted towards the camera. Therefore, the same surface will appear to have different temperature values when measured using a detector based on the micro-bolometry principle, which relies on resistance changes due to absorbed photon flux. This effect was experimentally observed and is discussed as follows.

The temperature of HL and CC patterned areas were measured and compared with a non-patterned area as the reference (R) summarised in Figure 6. For each monitored area, the area average temperature $T_{\mathrm{ave}}$ is defined as $T_{\mathrm{ave}}=\frac{1}{N}\sum_{i=1}^{N}T_{i}$, where *N* is the total number of pixels, and $T_{i}$ is the temperature of the *i* pixel. The thermal camera measures the intensity of IR radiation emitted from the surface and then determines the temperature by quantifying the amount of infrared emission it received in the spectral window 7.5–13 μm. According to Planck’s and Stefan–Boltzmann’s law [61], the higher temperature corresponds to higher IR emission from the sample. Thus, by measuring the temperature at different points on the sample’s surface, the thermal camera provides information about the thermal emissivity. This relationship between temperature and emitted power enables the estimation of the sample’s emissivity properties, as materials with higher emissivity will emit more IR radiation at a given temperature compared to those with lower emissivity. Figure 6c shows that laser-ablated gratings showed approximately (3–6)% larger temperature, demonstrating the grating pattern has a larger emissivity than a non-patterned area. There was also a slightly faster cooling (see dotted lines). However, the effect was within the uncertainty range.

The surface temperature was also measured with the same experimental setup at different azimuthal angles $\theta_{a}$ (Figure 7). The angular measurements were taken at an increment of $\theta_{a}=15^{{}^{\circ}}$, starting from 0° (top view) and reaching up to 75°. The measurement started when the sample temperature stabilised at 75 °C. The angular thermal emission was determined for different orientations of the linear and circular grating samples. A close to the Lambertial angular profile for emitted intensity $I\cos\theta_{a}$ was observed in the temperature determined from sample’s radiation (Figure 7). Geometrical anisotropy in patterns can influence the IR absorption due to orientation-sensitive scattering effects [62], which is also shown in the angular emission result. As discussed in Section 3.2, deeper grooves and more pronounced ripples formed when laser polarisation was perpendicular to the scanning direction (Figure 4(B1)) compared to when it was parallel to the scanning direction (Figure 4(B2)). As a result, the CC patterned area shows a higher angular emission at large azimuthal angles ($\theta_{a}>60^{{}^{\circ}}$), which is most obvious in Figure 7c. At small azimuthal angles ($\theta_{a}<45^{{}^{\circ}}$), the HL pattern is more dominant in IR emission. The orientation dependence is also shown by comparing the temperature difference of HL and CC patterns at small azimuthal angles, though the effect is not very pronounced. These optical anisotropy features were also studied in natural spider silk [63] and artificial nanomaterials [64,65].

## 4. Conclusions and Outlook

Fs laser ablation on sapphire was investigated for structure formation at different pulse fluence $F_{p}$ and the number of passes $N_{p}$. Spectral and thermal emission characteristics were analysed on 0.5 mm thick sapphire chips with period Λ=10 μm circular and linear gratings. The overall emissivity was significantly enhanced for both grating types. Such improvement also appears at 7–13 μm atmospheric transmission window, which is crucial for radiative cooling applications. The Lambertian nature of surface emitters was confirmed by measuring thermal emission (photon flux) at different azimuthal angles using an IR camera. Fs laser ablated gratings increased emissivity by (3–6)% compared to the bare sapphire surface.

Direct measurements of emissivity on laser-machined sapphire using both laboratory-based and synchrotron-based FTIR techniques were required to test long wavelengths in IR emissions and Reststrahlen bands, where Wolf’s effect and direction out-coupling of IR light were expected.

## Data Availability

All the data are presented within this manuscript.

## Appendix A. Four Polarisation R(λ) from Linear and Circular Al2O3 Gratings

Four-polarisation (4-pol.) method with linearly polarised incidence at four angles θ with $45^{{}^{\circ}}$ separation allows to define the orientation $\theta_{O}$ of the sample where the strongest absorption occurs from a fit $Amp\times \cos(2\theta +2\theta_{O})+Off$, where amplitude and offset of the fit are Amp,Off, respectively [66,67]. The 4-pol. method is applicable for far-field as well as near-field (non-propagating) [66,67]. Figure A1 has a summary of results, which reveal that reflectance *R* was orientationally isotropic for bare c-plance Al_2_O_3_ and circular grating pattern milled by laser ablation (a) and (c). Very strong modulation ΔR≈80% for linear grating was observed at λ=15.1 μm (inset on Figure A1b).

## Appendix B. Keldysh Parameter

The Keldysh parameter $\gamma_{K}$, serves as a crucial indicator to differentiate between the tunnelling or multi-photon ionisation when intense laser-matter interaction occurs, and it is calculated as [57,68,69,70]:

(A1) $$\gamma_{K}=\frac{\omega \sqrt{2mE_{g}}}{eE_{laser}}=\frac{\omega }{\omega_{t}},$$

here $E_{laser}$ [V/m] indicates electrical field strength at the peak intensity $I_{max}$, which is $2I_{p}$ for a Gaussian pulse with average intensity $I_{p}$, $E_{g}$ is the bandgap energy of material (8 eV is used as sapphire bandgap in the subsequent calculation), m,e are effective electron mass and electron charge, respectively, ω=2πc/λ is the cyclic frequency with λ being the central wavelength of ultra-short laser pulse, and the tunelling frequency is $\omega_{t}=eE_{laser}/\sqrt{2mE_{g}}$. This equation can be used when the bandgap is larger than the photon energy $\omega <E_{g}/\hbar$, where *ℏ* is Plank’s constant. The tunelling ionisation is dominant when $\gamma_{K}\ll 1$, that is a low frequency with high electrical field condition. On the other hand, the multi-photon absorption is dominant when $\gamma_{K}\gg 1$; in other words, a large frequency and moderate or low electrical field condition. When the frequency of the laser field is larger than the tunelling frequency $\omega >\omega_{t}$ the electron can tunnel through the barrier, and the tunelling becomes less efficient in material ionisation; $\omega_{t}\propto 1/time$ is defined by the time of flight of an electron through the potential barrier [69].

The $\gamma_{K}$ in Equation (A1) is estimated for this experiment with the average irradiance/intensity $I_{p}\approx$ 88 TW/cm^2^ (the Gaussian peak intensity $I_{max}=2I_{p}\approx$ 177 TW/cm^2^; Figure 4), the bandgap of sapphire $E_{g}=8$ eV and for wavelength λ=0.515 μm. One would find $\gamma_{K}=0.96<1$, which indicates a slight dominance of tunelling ionisation process.
